# Severe circumferential rectal ulcer associated with electric bidet toilet use

**DOI:** 10.1002/deo2.347

**Published:** 2024-03-07

**Authors:** Takuro Nishiwaki, Kenichiro Nakachi, Shin Inoue, Takashi Ashikawa, Tomoyuki Funato, Natsuki Kawamitsu, Shigenobu Yoshimura, Hironobu Nagumo, So Nakaji, Koichi Homma

**Affiliations:** ^1^ Department of Gastroenterology Kameda Medical Center Chiba Japan; ^2^ Department of Diagnostic Pathology Kameda Medical Center Chiba Japan

**Keywords:** colonoscopic findings, electric bidet toilet, excessive bidet use, rectal disorder, rectal ulcer

## Abstract

A 66‐year‐old man presented to the gastroenterology department with anal pain. For >10 years, he had used an electric bidet toilet while defecating for >5 min at a time, because of constipation. Two weeks prior to his visit, he became aware of discomfort in his anal area and had used an enema 1 week previously. He had persistent diarrhea and began to use the electric bidet toilet at the highest water pressure for long periods. As a result, his anal pain worsened. A colonoscopy revealed circumferential inflammation and ulceration extending from the anal canal to the lower rectum. Approximately half of the Japanese population washes their anuses before and after defecation. Cleaning the anus after defecation using a bidet contributes to hand hygiene and local comfort, and may be effective against constipation. However, excessive bidet use may cause rectal disorders, such as rectal mucosal prolapse syndrome and solitary rectal ulcers. Herein, we report a rare case of a patient with advanced rectal ulceration caused by electric bidet toilet usage.

## INTRODUCTION

Electric bidet toilets (EBTs) are sanitary devices that are integral to daily life and are widely used in Japan. Approximately half of the Japanese population washes their anus before and after defecation.^1^ Owing to the importance of bathroom hygiene and proper cleaning of soiled areas after bowel movement, the demand for EBTs has increased worldwide.^2^ Cleaning the anus after defecation using an EBT contributes to hand hygiene and local comfort; however, inappropriate EBT use can cause colonic and anal damage. Here, we report a case of a patient with severe rectal inflammation and ulceration caused by excessive bidet use.

## CASE REPORT

The patient was a 66‐year‐old man with a history of constipation. For >10 years, he used EBT for >5 min per session, to encourage defecation. Two weeks prior to the visit, he became aware of discomfort in his anal area and had used an enema one week previously. He had persistent diarrhea and worsening anal discomfort. He started using EBT at the maximum water pressure for long durations, which resulted in severe anal pain. Therefore, the patient visited the gastroenterology department.

His regular medications included magnesium oxide and sennosides. He had no history of anal intercourse. The patient had no recent history of overseas travel. His medical history included a left upper lobectomy that was used to treat left squamous cell carcinoma of the lung 5 years prior.

**FIGURE 1:**
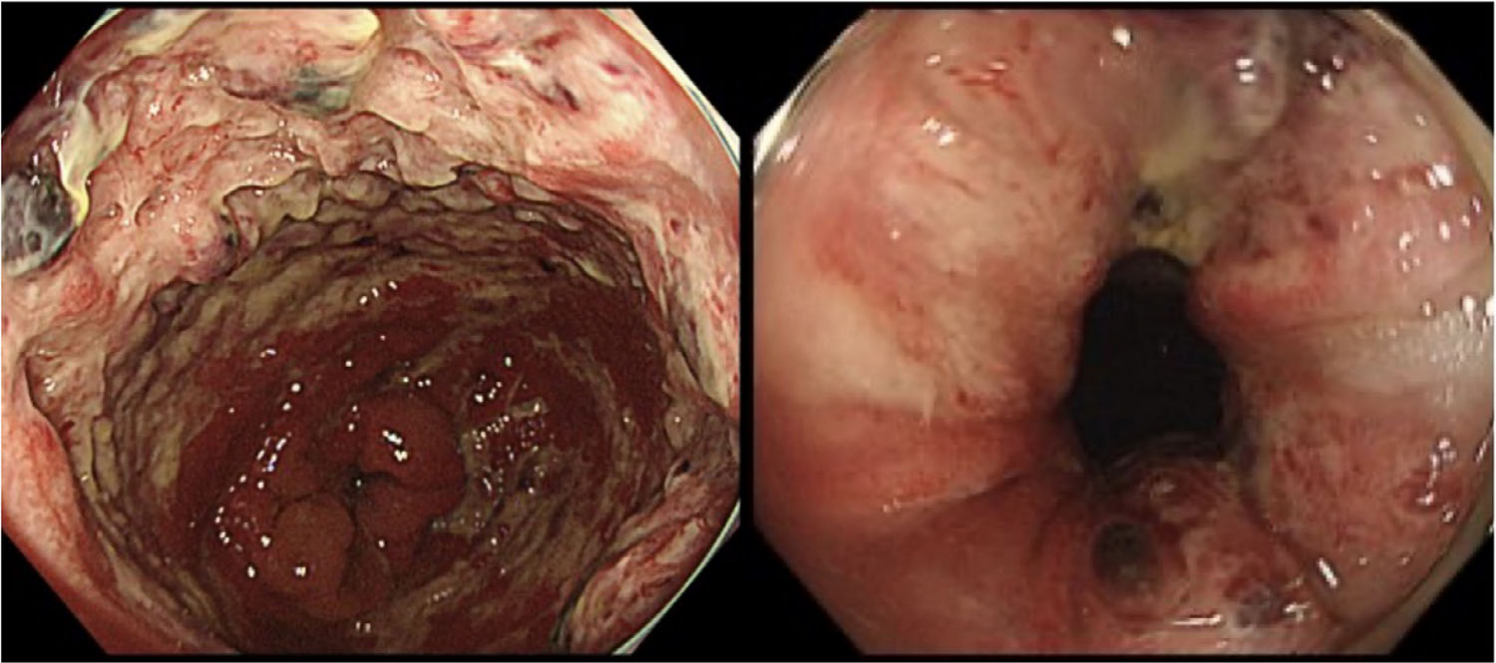
Colonoscopic findings at the diagnosis. Mucosal edema and ulceration with some adherent pus were observed circumferentially from the anal canal to the lower rectum.

**FIGURE 2:**
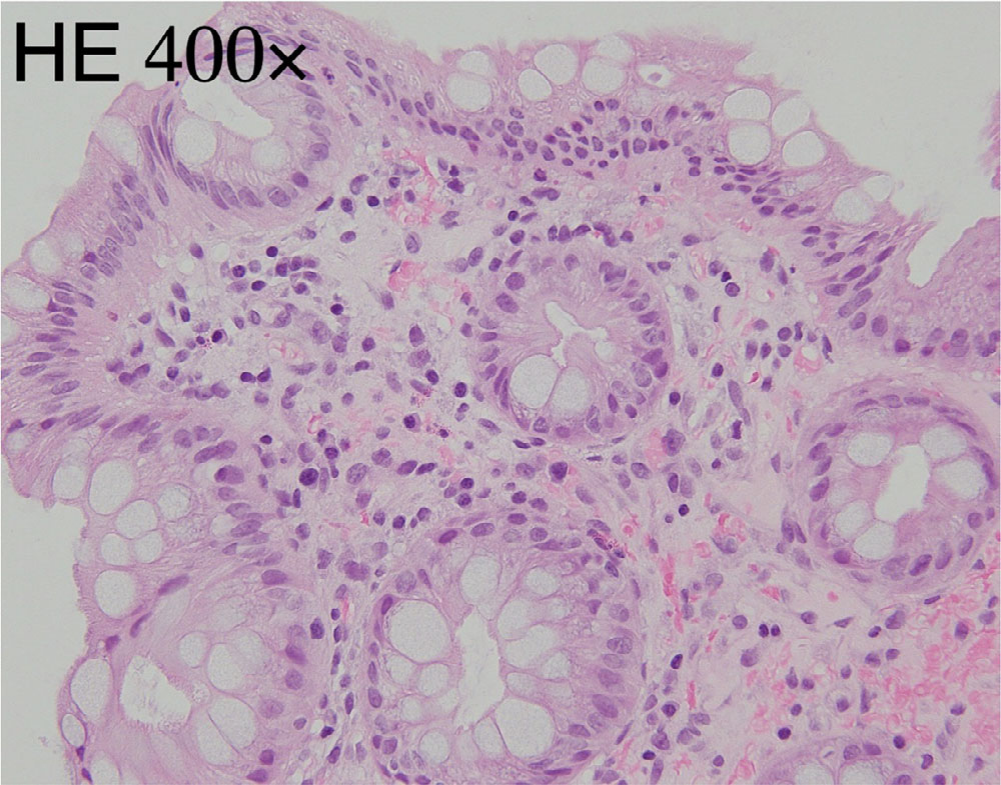
Histological images of the rectal mucosa. A non‐neoplastic rectal mucosa with mild inflammatory cellular infiltration and focal hemorrhaging was observed.

**FIGURE 3:**
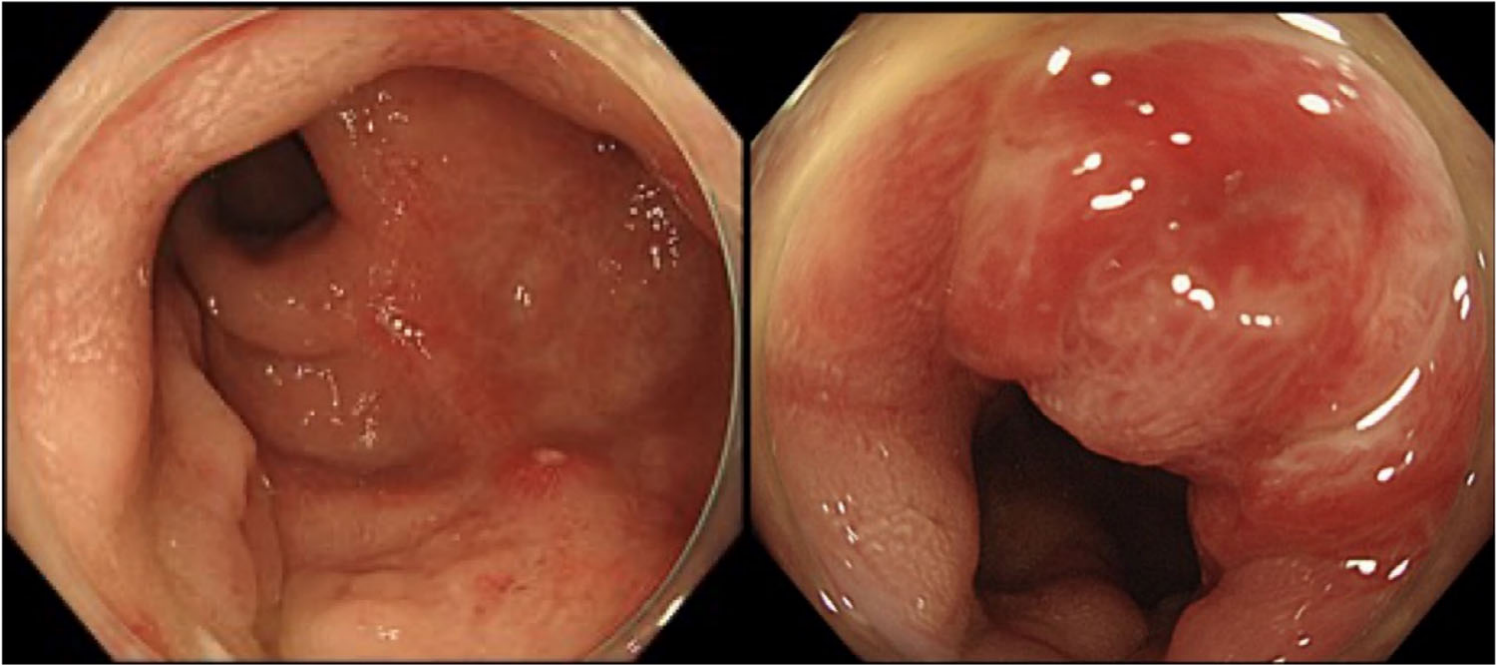
Colonoscopy at 2 months after the patient was instructed to discontinue electric bidet toilet use. Ulcer scars were observed in the rectum.

The physical examination revealed redness of the anus, and the rectal examination revealed a bloody mucus. An abdominal examination revealed no remarkable findings. A colonoscopy revealed circumferential mucosal edema and ulceration with pus adhering from the anal canal to the lower rectum. (Figure 1) Bacterial culture and tissue biopsy samples were obtained from the rectal mucosa, and *Aeromonas caviae*, *Escherichia coli*, and *Klebsiella pneumoniae* were detected. The histopathological examination revealed non‐neoplastic rectal mucosa with mild inflammatory cellular infiltration and localized hemorrhage. No nuclear inclusion bodies were detected in the biopsied specimen stained with hematoxylin and eosin (Figure 2). After instructing the patient to avoid EBT, his symptoms improved within approximately one week, and the discomfort in his anal area disappeared. The colonoscopy that was performed 2 months later revealed ulcer scars in the rectum (Figure 3).

## DISCUSSION

EBTs are common in Japan and have gradually become popular in other countries. EBTs contribute to hand hygiene^3^ and promote defecation. Bidet uses at low or medium water jet pressures and warm temperatures, and with a wide‐type water jet, potentially reduces anal pressure with an effect that resembles a warm sitz bath.^4^

In a survey of people who use EBTs to wash their anus before and after defecation, 70% of the respondents reported, “because stimulating the anus with a jet of water aids defecation,” and 20% reported, “because it aids defecation like an enema when water penetrates the rectum.”^5^ However, excessive bidet use may cause anal and rectal disorders, such as rectal mucosal prolapse syndrome and solitary rectal ulcers.^1^, ^6^ Anal disorders include anal pruritus, anal incontinence, and anal fissures. EBT with high water pressure has also been associated with rectal mucosal prolapse syndrome and direct solitary rectal ulcers.^2^, ^7^ The only reported case of rectal mucosal prolapse syndrome was excessive, prolonged, and repetitive straining of the EBT, causing the rectal mucosa to escape into the anal canal. Two cases of patients with rectal ulcers have been reported; both involved solitary ulcers that were thought to have been caused by damage to the rectal mucosa due to the direct application of water at high pressure.

In the present case, the patient presented with a severely circumscribed rectal ulcer, mucosal edema, and purulent discharge from the anal canal to the lower rectum. This type of case has never been seen before; therefore, we differentiated this case from ulcerative colitis and infectious enteritis at the time of colonoscopy.

Histopathology showed mild inflammatory cell infiltration of neutrophils and lymphocytes, but there were no signs of diffuse inflammatory cell infiltration, crypt abscesses, or severe goblet cell depletion, which are characteristics of ulcerative colitis. In contrast, the bacterial cultures detected *A. caviae*, *E. coli*, and *K. pneumoniae*. As *Aeromonas* spp. can cause bacterial enteritis, their involvement in the proctitis in this patient cannot be ruled out. Amoebic colitis was not suspected because there was no history of foreign travel or homosexuality, and antigen testing was not performed. No nuclear inclusion bodies were detected by hematoxylin and eosin staining alone.

The patient's medical history revealed that he had a habit of using bidets excessively and that he began to use them more excessively due to worsening diarrhea and anal pruritus he encountered after an enema. Specifically, he opened his anus and used a bidet at the maximum water pressure setting for approximately 10 min per session, more than five times a day, during the most recent week.

High‐pressure water can cause direct mechanical damage. In addition, the reflex contraction of the anal sphincter serves as an obstacle to defecation when a high‐pressure water jet is applied. Furthermore, the increase in anal sphincter pressure required to prevent water ingress might injure the mucosa and sphincter.^5^ In this case, the increased pressure on the anal sphincter due to the habit of using a bidet and the excessive increase in rectal pressure due to an enema a week earlier caused rectal injury, whereas the excessive use of the bidet caused direct mucosal damage. In addition, the mucosal edema and pus deposits that were noted on endoscopic findings, although still in the range of nonspecific enteritis, may have been caused by the hypotonicity of the water in the EBT, resulting in mucosal edema and possibly an infection. We believe that this full circumferential rectal ulcer was caused not only by direct damage from the water jet but also by the addition of chemical and infectious elements from the EBT water. The patient was successfully relieved of his symptoms upon discontinuing EBT use.

To avoid this problem, according to the Japan Restroom Manufacturers Association, a washing time of 10–20 s is suggested for use and should be observed.^8^ In addition, physicians should interview and instruct patients on the use of EBTs if a rectal ulcer or rectal mucosal change is detected during a colonoscopy procedure.

## CONFLICT OF INTEREST STATEMENT

None.

## ETHICS STATEMENT

All procedures followed have been performed in accordance with the ethical standards laid down in the Declaration of Helsinki and its later amendments.
